# Mendelian Randomization and Transcriptome-Wide Association Analysis Identified Genes That Were Pleiotropically Associated with Intraocular Pressure

**DOI:** 10.3390/genes14051027

**Published:** 2023-04-30

**Authors:** Zhikun Yang, Zhewei Zhang, Yining Zhu, Guangwei Yuan, Jingyun Yang, Weihong Yu

**Affiliations:** 1 Department of Ophthalmology, Peking Union Medical College Hospital, Key Laboratory of Ocular Fundus Diseases, Chinese Academy of Medical Sciences, Beijing 100730, China; 2 Department of Statistics, The Pennsylvania State University, State College, PA 16802, USA; 3 School of Mathematical Sciences, Fudan University, Shanghai 200433, China; 4 College of Professional Studies, Northeastern University, Boston, MA 02115, USA; 5 Rush Alzheimer’s Disease Center, Rush University Medical Center, Chicago, IL 60612, USA; jingyun_yang@rush.edu; 6 Department of Neurological Sciences, Rush University Medical Center, Chicago, IL 60612, USA

**Keywords:** intraocular pressure, expression quantitative trait loci, summary-based Mendelian randomization, genome-wide association study, transcriptome-wide association study

## Abstract

Background: Intraocular pressure (IOP) is a major modifiable risk factor for glaucoma. However, the mechanisms underlying the controlling of IOP remain to be elucidated. Objective: To prioritize genes that are pleiotropically associated with IOP. Methods: We adopted a two-sample Mendelian randomization method, named summary-based Mendelian randomization (SMR), to examine the pleiotropic effect of gene expression on IOP. The SMR analyses were based on summarized data from a genome-wide association study (GWAS) on IOP. We conducted separate SMR analyses using Genotype-Tissue Expression (GTEx) and Consortium for the Architecture of Gene Expression (CAGE) expression quantitative trait loci (eQTL) data. Additionally, we performed a transcriptome-wide association study (TWAS) to identify genes whose cis-regulated expression levels were associated with IOP. Results: We identified 19 and 25 genes showing pleiotropic association with IOP using the GTEx and CAGE eQTL data, respectively. *RP11-259G18.3* (P_SMR_ = 2.66 × 10^−6^), *KANSL1-AS1* (P_SMR_ = 2.78 × 10^−6^), and *RP11-259G18.2* (P_SMR_ = 2.91 × 10^−6^) were the top three genes using the GTEx eQTL data. *LRRC37A4* (P_SMR_ = 1.19 × 10^−5^), *MGC57346* (P_SMR_ = 1.19 × 10^−5^), and *RNF167* (P_SMR_ = 1.53 × 10^−5^) were the top three genes using the CAGE eQTL data. Most of the identified genes were found in or near the 17q21.31 genomic region. Additionally, our TWAS analysis identified 18 significant genes whose expression was associated with IOP. Of these, 12 and 4 were also identified by the SMR analysis using the GTEx and CAGE eQTL data, respectively. Conclusions: Our findings suggest that the 17q21.31 genomic region may play a critical role in the regulation of IOP.

## 1. Introduction

Intraocular pressure (IOP) refers to the fluid pressure inside the eye, which is affected by the production and drainage of the aqueous humor. Excessive production or insufficient drainage of aqueous humor can lead to ocular hypertension (OHT), which has a prevalence of approximately 1.6% in the general population over 30 years old [1]. The prevalence of OHT can be higher in persons over 40 years old, ranging from 2.6% to 3.6% [2,3,4]. IOP can be modified by various treatments, such as eye drops, laser, and surgery [5]. Previous studies have shown that each 1 mm Hg drop of IOP can lower the risk of glaucoma progression by 10% [6,7]. Therefore, IOP represents a major modifiable risk factor of glaucoma [8], the most common cause of irreversible blindness worldwide [9]. 

Previous research indicated that both environmental and genetic factors affect IOP [10]. Smoking, alcohol consumption, and dietary omega-3 fatty acids have shown epidemiological associations with primary open-angle glaucoma (POAG) [11,12,13]. Twin and family studies have estimated the heritability of IOP to be between 40% and 70% [14,15]. Multiple genetic variants, mutations, and genomic loci have been found to be associated with IOP [10,16,17,18,19,20,21,22,23,24,25,26,27]. Despite these informative findings, the mechanisms underlying the control of aqueous humor dynamics and IOP regulation are still poorly understood. More studies are needed to explore the complex mechanisms underlying the regulation of IOP.

In this paper, we investigated genes that are pleiotropically associated with IOP by using a recently developed two-sample Mendelian randomization (MR), named summary-based Mendelian randomization (SMR), that integrates genome-wide association study (GWAS) summary data for IOP and cis-eQTL (expression quantitative trait loci) data. We also conducted a transcriptome-wide association study (TWAS) to identify genes whose cis-regulated expression levels are associated with IOP. 

## 2. Methods

### 2.1. Editorial Policies and Ethical Considerations

**Figure 1:**
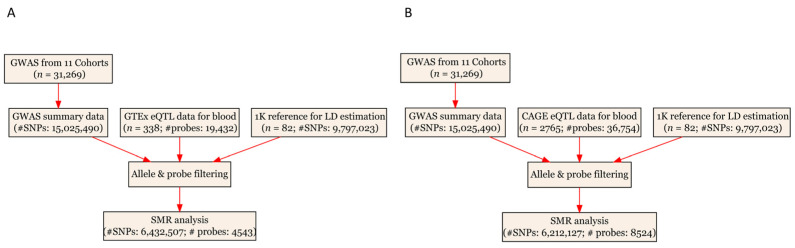
Flow chart for the SMR analyses. (**A**) SMR analysis using GTEx eQTL data for blood; and (**B**) SMR analysis using CAGE eQTL data for blood. CAGE, Consortium for the Architecture of Gene Expression; eQTL, expression quantitative trait loci; GWAS, genome-wide association studies; GTEx, Genotype-Tissue Expression; LD, linkage disequilibrium; IOP, intraocular pressure; SMR, summary-based Mendelian randomization; SNP, single nucleotide polymorphisms.

This study utilized GWAS summary results for IOP and eQTL data. All the data were publicly available. As a result, ethical considerations are not needed. The analytical process of the present study is illustrated in Figure 1.

### 2.2. GWAS Data for IOP

The GWAS summary data for IOP were provided by a recent multi-trait genome-wide association meta-analysis of optic disc parameters [28]. The results were based on genetic data imputed using the Haplotype Reference Consortium (HRC) and included 11 cohorts from the International Glaucoma Genetics Consortium (IGGC). A total of 31,269 participants of European ancestry were included in the meta-analysis. An additive genetic model was assumed by all the participating studies, adjusting for age, sex, and at least the first five principal components, as well as cohort-specific covariates when necessary. The GWAS summarized data are publicly available and can be downloaded from http://ftp.ebi.ac.uk/pub/databases/gwas/summary_statistics/GCST009001-GCST010000/GCST009413 (accessed on 26 July 2021).

### 2.3. eQTL Data

The SMR analyses utilized cis-eQTL genetic variants as instrumental variables (IVs) for gene expression. Because eQTL data for the eye were unavailable, eQTL data for blood from Genotype-Tissue Expression (GTEx) and Consortium for the Architecture of Gene Expression (CAGE) were used for the SMR analyses. The V7 release of the GTEx eQTL data for blood [29] included 338 participants, while the CAGE eQTL data for blood [30] included 2765 participants. The eQTL data can be downloaded at https://cnsgenomics.com/data/SMR/#eQTLsummarydata (accessed on 26 July 2021).

### 2.4. SMR Analysis

The Mendelian analyses were conducted using the method implemented in the SMR software version 1.3.1 [31]. SMR applies the principles of MR, integrating GWAS and eQTL summary statistics to explore the pleiotropic association between gene expression and a trait. In SMR, genetic variants are used as the IVs, and estimation of the pleiotropic association can be made because the inherited genetic variants are independent of potentially confounding factors [32]. Here, ‘pleiotropic association’ between gene expression and IOP refers to either pleiotropy (i.e., both gene expression and IOP are affected by the same causal variant) or causality (i.e., the effect of a causal variant on IOP is mediated by gene expression). In either case, gene expression is the exposure but not an outcome (i.e., SNP > gene expression). SMR is not designed to examine whether a trait influences gene expression.

The SMR analysis followed a similar approach as described in a previous publication [33], using all the default settings in SMR software. Details about the settings adopted in the SMR analyses can be found in Table S1. The multiple testing was adjusted by the false discovery rate (FDR).

### 2.5. TWAS Analysis

We also conducted a TWAS analysis [34] integrating the GWAS summary statistics of IOP and pre-computed gene expression weights to further explore genes whose cis-regulated expression is associated with IOP. This approach imputed gene expression from summarized GWAS data to test its association with the phenotype of interest. The effect size of the expression of a gene for a trait can be viewed as a linear combination of genetic effects on a trait, with weights calculated based on the correlation between SNPs and gene expression while accounting for linkage disequilibrium (LD) among the SNPs. The weights are generally pre-computed using data from a relatively small set of reference individuals for whom both gene expression and genetic variations (SNPs) are available [34]. Although this TWAS approach is conceptually similar to SMR, it is less strict than SMR in that it aims to identify genes whose genetically controlled expression is associated with a disease, while SMR aims to identify genes that are pleiotropically associated with a disease. As we observed multiple significant genes located in 17q21.31, for this risk locus, we performed joint/conditional analysis, a process to identify which genes represent independent associations (i.e., jointly significant), and which genes are not significant after accounting for the predicted expression of other genes in the region (i.e., marginally significant). The analyses were performed by using FUSION software. We applied the weights that were pre-computed from the GTEx v7 whole-blood reference expression panel and adopted all the default settings in FUSION.

The data curation and statistical/bioinformatical analysis were performed using R version 4.1.1, R Foundation for Statistical Computing, Vienna, Austria (https://www.r-project.org/, accessed on 27 April 2023), PLINK 1.9 (https://www.cog-genomics.org/plink/1.9/, accessed on 27 April 2023), SMR version 1.3.1 (https://yanglab.westlake.edu.cn/software/smr, accessed on 27 April 2023), and FUSION (http://gusevlab.org/projects/fusion/, accessed on 27 April 2023).

## 3. Results

### 3.1. Basic Information of the Summarized Data

The CAGE eQTL data have a much larger number of participants compared to the GTEx eQTL data (2765 vs. 338), as well as a larger number of eligible probes (8524 vs. 4543). After checking the allele frequencies between the datasets and performing LD pruning, there were more than 6.2 million eligible SNPs in each SMR analysis (Table 1).

### 3.2. Pleiotropic Association with IOP

**Figure 2:**
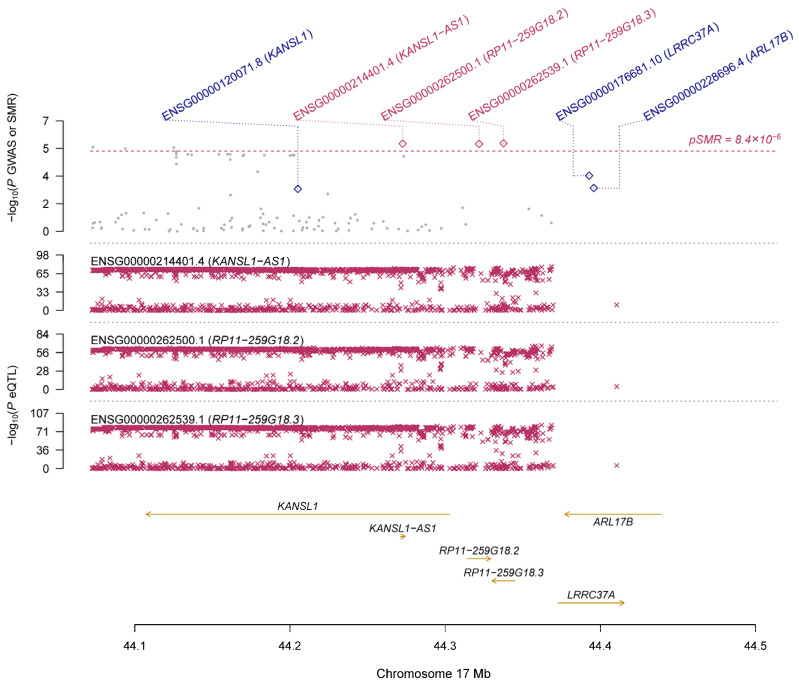
Pleiotropic association of *RP11-259G18.3*, *KANSL1-AS1*, and *RP11-259G18.2* with IOP. Top plot, grey dots represent the −log10 (*p* values) for SNPs from the GWAS of IOP, with solid rhombuses indicating that the probes pass the HEIDI test. Middle plot, eQTL results. Bottom plot, location of genes tagged by the probes. GWAS, genome-wide association studies; SMR, summary-based Mendelian randomization; HEIDI, heterogeneity in dependent instruments; eQTL, expression quantitative trait loci; IOP, intraocular pressure.

**Figure 3:**
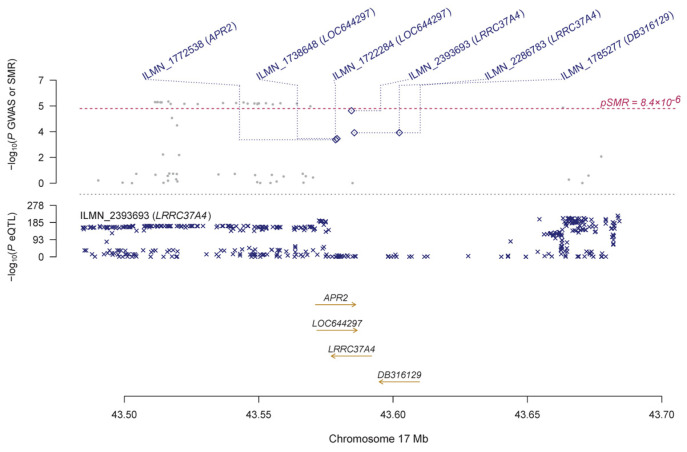
Pleiotropic association of *LRRC37A4* with IOP. Top plot, grey dots represent the −log10 (*p* values) for SNPs from the GWAS of IOP, with solid rhombuses indicating that the probes pass the HEIDI test. Middle plot, eQTL results. Bottom plot, location of genes tagged by the probes. GWAS, genome-wide association studies; SMR, summary-based Mendelian randomization; HEIDI, heterogeneity in dependent instruments; eQTL, expression quantitative trait loci; IOP, intraocular pressure.

**Figure 4:**
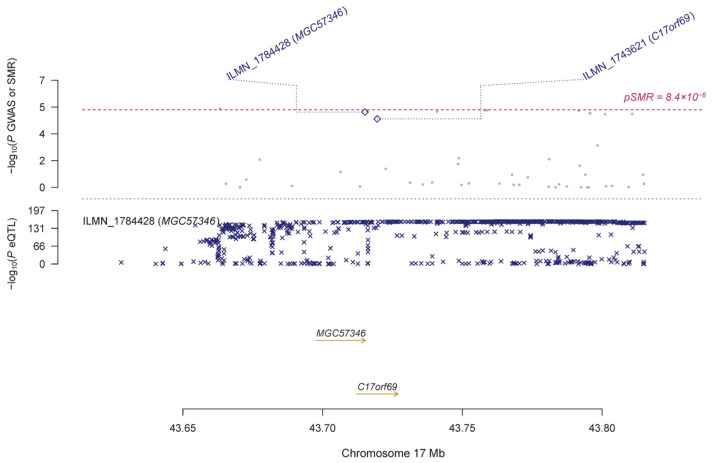
Pleiotropic association of *MGC57346* with IOP. Top plot, grey dots represent the −log10 (*p* values) for SNPs from the GWAS of IOP, with solid rhombuses indicating that the probes pass the HEIDI test. Middle plot, eQTL results. Bottom plot, location of genes tagged by the probes. GWAS, genome-wide association studies; SMR, summary-based Mendelian randomization; HEIDI, heterogeneity in dependent instruments; eQTL, expression quantitative trait loci; IOP, intraocular pressure.

**Figure 5:**
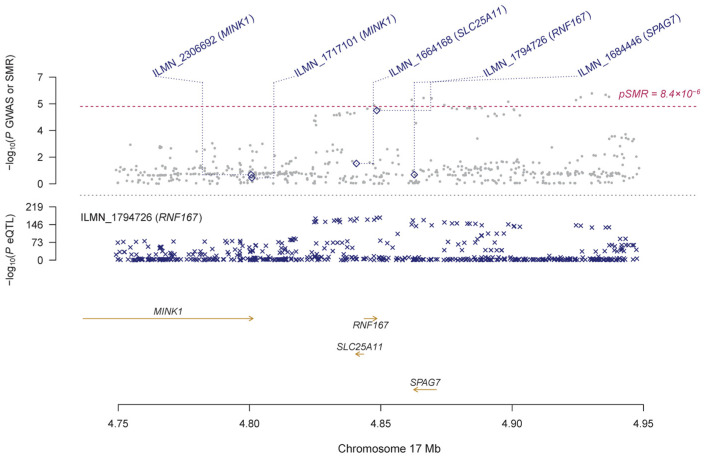
Pleiotropic association of *RNF167* with IOP. Top plot, grey dots represent the −log10 (*p* values) for SNPs from the GWAS of IOP, with solid rhombuses indicating that the probes pass the HEIDI test. Middle plot, eQTL results. Bottom plot, location of genes tagged by the probes. GWAS, genome-wide association studies; SMR, summary-based Mendelian randomization; HEIDI, heterogeneity in dependent instruments; eQTL, expression quantitative trait loci; IOP, intraocular pressure.

Using the GTEx eQTL data, our SMR analysis identified 19 genes that are pleiotropically associated with IOP (Table 2 and Table S2), with *RP11-259G18.3* (ENSG00000262539.1; β [SE] = 0.12 [0.03], P_SMR_ = 2.66 × 10^−6^; Figure 2), *KANSL1-AS1* (ENSG00000214401.4; β [SE] = 0.13 [0.03], P_SMR_ = 2.78 × 10^−6^; Figure 2), and *RP11-259G18.2* (ENSG00000262500.1; β [SE] = 0.14 [0.03], P_SMR_ = 2.91 × 10^−6^; Figure 2) being the top three genes. Most of the identified genes are located on chromosome 17, except for a few such as *ABO* (ENSG00000175164.9, β [SE] = 0.21 [0.05], P_SMR_ = 6.40 × 10^−6^; Supplementary Figure S1). Using the CAGE eQTL data, our SMR analysis identified 25 unique genes that are pleiotropically associated with IOP (Table 2 and Table S3), with *LRRC37A4* (ILMN_2393693; β [SE] = −0.12 [0.03], P_SMR_ = 1.19 × 10^−5^; Figure 3), *MGC57346* (ILMN_1784428; β [SE] = 0.15 [0.03], P_SMR_ = 1.19 × 10^−5^; Figure 4), and *RNF167* (ILMN_1794726; β [SE] = 0.15 [0.03], P_SMR_ = 1.53 × 10^−5^; Figure 5) being the top three genes. Similarly, most of the identified genes are located on chromosome 17, except for a few such as *SGTB* (ILMN_2109343; β [SE] = −0.16 [0.04], P_SMR_ = 3.45 × 10^−5^; Supplementary Figure S2). Four genes, including *DAB2*, *LOC644297*, *LRRC37A4*, and *NSF*, were each tagged by two probes.

### 3.3. Cis-Regulated Gene Expression in Association with IOP

**Figure 6:**
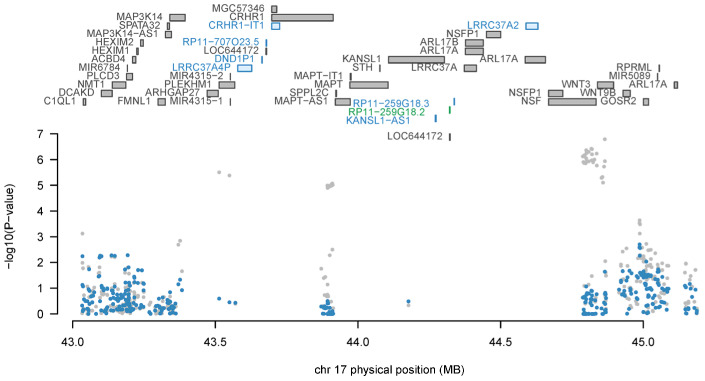
Joint/conditional analysis of TWAS significant loci on 17q21.31. The top panel of the joint/conditional plot displays all genes that are in the loci (usually gray), with marginally significant TWAS association genes highlighted in blue, and jointly significant genes in green. The bottom panel is the Manhattan plot of the original GWAS summary statistics data before (gray) and after (blue) conditioning on the green genes. TWAS, transcriptome-wide association study; GWAS, genome-wide association study.

We found 18 significant genes whose expression was associated with IOP by FUSION analysis after correction for multiple testing (FDR < 0.05; Table S4), with *RP11-259G18.2* (ENSG00000262500.1; Z = 4.87, P_TWAS_ = 1.13 × 10^−6^), *RP11-259G18.3* (ENSG00000262539.1; Z = 4.85, P_TWAS_ = 1.26 × 10^−6^), and *NUP160* (ENSG00000030066.9; Z = −4.62, P_TWAS_ = 3.83 × 10^−6^) being the top three genes. Most of the significant genes are located on chromosome 17. Of the 18 significant genes, 12 were also identified by the SMR analysis using the GTEx eQTL data, including *RP11-259G18.2*, *RP11-259G18.3*, *ABO*, *CRHR1-IT1*, *KANSL1-AS1*, *RP11-707O23.5*, *LRRC37A2*, *DND1P1*, *LRRC37A4P*, *AFAP1*, *TEF*, and *MEI1*. Four were identified by the SMR analysis using the CAGE eQTL data, including *LRRC37A2*, *AFAP1*, *TEF*, and *MEI1*. Therefore, these four genes were identified by FUSION and the two SMR analyses (Table S4). The joint/conditional tests for 17q21.31 indicated that *RP11−259G18.2* was jointly significant (i.e., independent association), and seven genes were marginally significant, including *RP11-259G18.3*, *CRHR1-IT1*, *KANSL1-AS1*, *RP11-707O23.5*, *LRRC37A2*, *DND1P1*, and *LRRC37A4P* (Figure 6).

## 4. Discussion

In this study, we identified multiple genes showing a pleiotropic association with IOP through SMR and TWAS approaches. Our results confirmed findings from previous studies and revealed novel genes related to IOP regulation.

In our SMR analyses, the IVs were based on eQTL data, and the exposure was (transcriptome-wide) gene expression. The GWAS used genetic data imputed based on the HRC and included 11 cohorts from the International Glaucoma Genetics Consortium (IGGC) [28]. The GTEx eQTL data were based on deceased donors [29], and the CAGE eQTL data were based on samples from five cohorts [30]. These cohorts were not part of the GWAS summary results for IOP. Therefore, there is no overlap between the samples. MR can be conducted based on data from one sample or two samples. The summary association results came from the same individuals in the one-sample MR, and from different, potentially overlapping sets of individuals in the two-sample MR [35]. We chose the ‘two-sample MR’ over the ‘one-sample MR’ for several reasons: (1) The eQTL data are unavailable for the subjects in the GWAS data; (2) using the association results from the same or partially overlapping samples may introduce weak instrument bias [35]; and, (3) the power of an MR can be greatly increased by using a two-sample MR approach [36,37].

In our study, most of the genes showing a pleiotropic association with IOP are near 17q21.31 (chr17: 40900001-44900000, GRCh37/hg19), a structurally complex and evolutionarily dynamic region of the genome [38,39,40]. This region contains a ~970 kb inversion of the *MAPT* locus in populations of European ancestry [41]. *MAPT* (microtubule-associated protein tau) is associated with both the ganglion cell inner plexiform layer (GCIPL) and the retinal nerve fiber layer (RNFL), indicating that it might impact glaucoma pathogenesis through modulation of retinal thickness [42]. The *MAPT* locus has two divergent haplotypes, H1 (direct orientation) and H2 (inverted orientation), with distinct functional impacts [38]. Although both GTEx and CAGE do have eQTL data for *MAPT*, it was dropped in the SMR analysis because SMR only includes cis-eQTL with a *p*-value < 5 × 10^−8^ (The minimum *p*-value is 1.41 × 10^−4^ for cis-eQTL in the GTEx and 2.62 × 10^−7^ in the CAGE). Despite that, some of the genes identified in our study were reported to be associated with *MAPT* haplotypes. For example, *LRRC37A4* is the top-hit gene in the SMR analysis using the CAGE eQTL data, and the H1 haplotype of *MAPT* is associated with an increased expression of it [41]. Moreover, several other identified genes in the 17q21.31 region are either associated with IOP or act collectively in influencing IOP or associated traits. For instance, a GWAS identified 139 genetic loci associated with the macular thickness (MT), including genetic variants in *KANSL1*, *LRRC37A4P*-MAPK8IP1P2, and *NSF* [43]. In addition, *KANSL1-AS1* (identified in GTEx), *LRRC37A2* (identified in CAGE), and *OR7E14P* were found to form a regulatory cluster in influencing both IOP and MT [44]. Together, these findings suggest that 17q21.31 is crucial for IOP regulation. However, the exact pathogenesis remains unclear. Further investigation is needed to elucidate the exact functions of this region and examine its biological role in influencing IOP and the pathogenesis of glaucoma.

We found a significant pleiotropic association between *ABO* and IOP using GTEx eQTL data. *ABO* (Alpha 1-3-N-Acetylgalactosaminyltransferase and Alpha 1-3-Galactosyltransferase) is located on chromosome 9q34.2 and encodes proteins related to the first discovered blood group system [45]. It has seven exons and six introns [46]. A variation in *ABO* forms the basis of the ABO blood group [47]. Genetic variants in *ABO* are associated with various health conditions, such as diabetes, thromboembolism, myocardial infractions, atherosclerosis, and stroke [48,49]. In a previous multi-ancestry meta-analysis, the International Glaucoma Genetics Consortium (IGGC) revealed a novel *ABO* polymorphism (rs8176693) associated with IOP [50]. A later meta-analysis showed that the *ABO* polymorphism rs8176741 was significantly associated with IOP, vertical cup–disc ratio (VCDR), and cup area [51]. Despite these encouraging findings, the exact mechanisms underlying the observed association between genetic variants in *ABO* and IOP remain to be elucidated. More research is needed to understand the functions and roles of *ABO* in influencing IOP.

In our study, we found that some genes, such as *AFAP1*, showed a pleiotropic association with IOP. *AFAP1* (Actin Filament Associated Protein 1) is located on 4p16.1 and encodes a protein that binds and crosslinks filaments [52,53]. Actin cytoskeleton-modulating signals are involved in the regulation of aqueous outflow and IOP [54,55]. Two SNPs in *AFAP1* (rs4619890 and rs11732100) have been reported to be associated with POAG in GWAS studies [56,57]. In European-ancestry populations, the two SNPs are moderately associated with another SNP in *AFAP1* (rs28795989) linked to IOP [25]. Another GWAS found that rs6816389 in *AFAP1* is associated with IOP in European-ancestry participants [58]. Moreover, the expression of *AFAP1* was detected in the trabecular meshwork, retina (including retinal ganglion cells [RGCs]), and optic nerve of a normal human eye and a glaucomatous eye [57]. Together, existing evidence implies *AFAP1*’s potential involvement in the pathogenesis of glaucoma. 

We also identified several other genes that are not on 17q21.31 but show a significant pleiotropic association with IOP regulation, such as *SGTB* and *TEF*. The *SGTB* gene, also known as the small glutamine-rich tetratricopeptide repeat (TPR)-containing beta, is located on 5q12.3 and belongs to the SGT (small glutamine-rich TPR-containing protein) family. SGT proteins have been associated with a variety of biological processes, including neuronal synaptic transmission, cell cycle regulation, protein folding, and apoptosis [59,60,61]. Previous research discovered that SGTB interacts with Brother of CDO (BOC) and modulates its surface presence. This subsequently leads to JNK activation, which, in turn, facilitates neuronal differentiation and the growth of neurites [62]. It was found that genetic variants near *SGTB* are related to IOP or corneal thickness (CCT) [63,64]. The *TEF* gene, also known as thyrotroph embryonic factor, is located on 22q13.2. The TEF protein belongs to the PAR (proline and acidic amino acid-rich) subfamily of bZIP transcription factors [65]. The proteins encoded by these genes have recently been demonstrated to regulate the expression of many enzymes and molecules involved in detoxification and drug metabolism [8]. A previous study found that the genetic polymorphism rs6519240 near *TEF* is associated with refractive error [66]. However, studies on the relationship between *SGTB*, *TEF*, and IOP regulation are scarce. Further investigation is necessary to elucidate the role of these genes in IOP regulation.

An SMR analysis relies on three key assumptions. First, the genotype is associated with gene expression. Second, confounding factors that bias the associations between gene expression and IOP are not associated with the genotype. Third, the genotype is related to IOP only through its association with gene expression. For assumption 1, we used the default *p*-value threshold of 5 × 10^−8^ to select the top associated eQTL in our SMR analyses. Therefore, the genetic variants selected as IVs are indeed strongly associated with gene expression, and weak instruments are unlikely to be a big concern. Assumption 2 is difficult to verify directly, as SMR analyses use summarized data. The assumption is often based on the biological belief that genotypes are unrelated to confounding factors, such as socioeconomics and behavioral characteristics [32]. For assumption 3, horizontal pleiotropy was found in about half of the significant causal relationships in MR, which could introduce distortions as high as 201% in the causal estimates. Horizontal pleiotropy could induce false-positive causal findings in up to 10% of the relationships [67]. We observed some pleiotropic associations with significant HEIDI tests, suggesting horizontal pleiotropy (Table 2). Therefore, caution should be exercised when interpreting the corresponding findings.

Our study has limitations. The eQTL data are based on limited sample sizes, which may affect the statistical power. Additionally, the eQTL data have limited eligible probes. As a result, we may have missed some important genes. Despite this, the power of SMR has been examined through extensive simulation studies. The simulations showed that SMR was equivalent to MR analysis if the genotype, gene expression, and phenotype data were from the same sample. The power of SMR could be greatly increased if the eQTL data and GWAS summary results were from two independent samples with very large sample sizes [31]. We believe that concerns about the power of our SMR analyses are minimal, especially in the SMR analysis using the CAGE eQTL data. The SMR approach cannot differentiate between pleiotropy and causality. We used eQTL data from blood because usable eQTL data from the eye are unavailable. Future studies using eye eQTL data are needed to validate our findings. Our SMR analyses used data from participants of European ancestry. Since the prevalence of POAG is ethnicity-specific, it is reasonable to postulate that the GWAS results might also be ethnicity-specific. Therefore, our results might not be generalized to other ethnic populations.

## 5. Conclusions

We identified several genes that are pleiotropically associated with IOP. Our results indicate that the 17q21.31 genomic region could be crucial for IOP regulation. Future studies are necessary to clarify the collective actions of the genes identified in the 17q21.31 genomic region and the roles of the identified genes on the other chromosomes in the IOP regulation.

## Figures and Tables

**Table 1:** Basic information on the eQTL and GWAS data.

Data Source	Total Number of Participants	Number of Eligible Genetic Variants or Probes
SMR using GTEx eQTL data		
eQTL	338	4543
GWAS	31,269	6,432,507
SMR using CAGE eQTL data		
eQTL	2765	8524
GWAS	31,269	6,212,127

CAGE, Consortium for the Architecture of Gene Expression; eQTL, expression quantitative trait loci; GWAS, genome-wide association studies; GTEx, Genotype-Tissue Expression; SMR, summary-based Mendelian randomization.

**Table 2:** The top ten genes showing significant pleiotropic association with IOP.

eQTL Data	Probe	Gene	CHR	Top SNP	P_eQTL_	P_GWAS_	Beta	SE	P_SMR_	P_HEIDI_	Q Value
**GTEx**	ENSG00000214401.4	*KANSL1-AS1*	17	rs199534	2.42 × 10^−76^	1.24 × 10^−6^	0.125	0.027	2.78 × 10^−6^	0.022	1.84 × 10^−5^
	ENSG00000262500.1	*RP11-259G18.2*	17	rs199451	2.19 × 10^−66^	1.19 × 10^−6^	0.141	0.030	2.91 × 10^−6^	0.049	1.84 × 10^−5^
	ENSG00000262539.1	*RP11-259G18.3*	17	rs199534	5.96 × 10^−81^	1.24 × 10^−6^	0.120	0.026	2.66 × 10^−6^	0.046	1.84 × 10^−5^
	ENSG00000175164.9	*ABO*	9	rs12216891	1.20 × 10^−22^	3.70 × 10^−7^	0.208	0.046	6.40 × 10^−6^	0.412	3.04 × 10^−5^
	ENSG00000264070.1	*DND1P1*	17	rs112578465	1.20 × 10^−60^	5.00 × 10^−6^	0.120	0.027	1.07 × 10^−5^	0.370	4.08 × 10^−5^
	ENSG00000214425.2	*LRRC37A4P*	17	rs79501144	1.11 × 10^−102^	1.04 × 10^−5^	−0.115	0.027	1.57 × 10^−5^	0.526	4.50 × 10^−5^
	ENSG00000263503.1	*RP11-707O23.5*	17	rs111273167	1.88 × 10^−75^	1.08 × 10^−5^	0.118	0.028	1.90 × 10^−5^	0.530	4.50 × 10^−5^
	ENSG00000204650.9	*CRHR1-IT1*	17	rs1724390	1.21 × 10^−53^	7.53 × 10^−6^	0.251	0.058	1.66 × 10^−5^	0.893	4.50 × 10^−5^
	ENSG00000238083.3	*LRRC37A2*	17	rs17426174	6.85 × 10^−38^	1.63 × 10^−5^	0.161	0.039	4.28 × 10^−5^	0.002	9.04 × 10^−5^
	ENSG00000196526.6	*AFAP1*	4	rs62290601	7.45 × 10^−80^	8.11 × 10^−5^	−0.096	0.025	1.12 × 10^−4^	0.030	2.12 × 10^−4^
**CAGE**	ILMN_1794726	*RNF167*	17	rs238243	2.22 × 10^−175^	1.23 × 10^−5^	0.147	0.034	1.53 × 10^−5^	0.627	1.48 × 10^−4^
	ILMN_2393693	*LRRC37A4*	17	rs113661667	5.03 × 10^−228^	9.86 × 10^−6^	−0.124	0.028	1.19 × 10^−5^	0.003	1.48 × 10^−4^
	ILMN_1784428	*MGC57346*	17	rs62057067	2.43 × 10^−157^	8.99 × 10^−6^	0.146	0.033	1.19 × 10^−5^	0.010	1.48 × 10^−4^
	ILMN_1680353	*NSF*	17	rs199442	5.94 × 10^−15^	3.84 × 10^−7^	0.612	0.144	2.11 × 10^−5^	0.108	1.53 × 10^−4^
	ILMN_2109343	*SGTB*	5	rs42884	4.56 × 10^−137^	2.68 × 10^−5^	−0.159	0.038	3.45 × 10^−5^	0.015	1.67 × 10^−4^
	ILMN_1743621	*C17orf69*	17	rs113029914	8.60 × 10^−34^	1.01 × 10^−5^	0.334	0.081	3.32 × 10^−5^	0.005	1.67 × 10^−4^
	ILMN_1701998	*AFAP1*	4	rs62290601	1.26 × 10^−179^	8.11 × 10^−5^	−0.127	0.033	9.23 × 10^−5^	0.009	3.35 × 10^−4^
	ILMN_2330845	*NSF*	17	rs199446	1.03 × 10^−10^	7.35 × 10^−7^	0.736	0.187	8.50 × 10^−5^	0.257	3.35 × 10^−4^
	ILMN_1706511	*TEF*	22	rs4822025	1.02 × 10^−27^	5.19 × 10^−5^	0.324	0.085	1.49 × 10^−4^	0.381	4.80 × 10^−4^
	ILMN_1737195	*CENPK*	5	rs154940	3.27 × 10^−299^	1.58 × 10^−4^	−0.100	0.027	1.75 × 10^−4^	0.002	5.09 × 10^−4^

The GWAS summarized data were provided by the study of Bonnemaijer et al. [28] and can be downloaded at http://ftp.ebi.ac.uk/pub/databases/gwas/summary_statistics/GCST009001-GCST010000/GCST009413 (accessed on 26 July 2021). The CAGE and GTEx eQTL data can be downloaded at https://cnsgenomics.com/data/SMR/#eQTLsummarydata (accessed on 26 July 2021). Only the top ten significant results are shown for each SMR analysis. For the complete significant results, please see Tables S1 and S2. Top SNP means the top cis-eQTL and was used as the instrument variable. P_eQTL_ is the *p*-value of the top associated cis-eQTL in the eQTL analysis, and P_GWAS_ is the *p*-value for the top associated cis-eQTL in the GWAS analysis. Beta is the estimated effect size in the SMR analysis, SE is the corresponding standard error, P_SMR_ is the *p*-value for the SMR analysis, P_HEIDI_ is the *p*-value for the HEIDI test, and the Q value is the adjusted *p*-value found using FDR. The FDR was calculated at *p* = 10^−3^ threshold. CHR, chromosome; HEIDI, heterogeneity in dependent instruments; IOP, intraocular pressure; SNP, single-nucleotide polymorphism; SMR, summary-based MendelianrRandomization; QTL, quantitative trait loci; FDR, false discovery rate; GWAS, genome-wide association studies.

## Data Availability

All the data generated or analyzed during this study are publicly available as specified in Section 2 of this paper. Specifically, the eQTL data can be downloaded at https://cnsgenomics.com/data/SMR/#eQTLsummarydata (accessed on 26 July 2021), and the GWAS summarized data can be downloaded at http://ftp.ebi.ac.uk/pub/databases/gwas/summary_statistics/GCST009001-GCST010000/GCST009413 (accessed on 26 July 2021).

## Supplementary Materials

The following supporting information can be downloaded at: https://www.mdpi.com/article/10.3390/genes14051027/s1, Figure S1: Pleiotropic association of *ABO* with IOP; Figure S2: Pleiotropic association of *SGTB* with IOP; Table S1: Default parameters in the SMR analyses; Table S2: Genes showing significantly pleiotropic association with IOP using GTEx eQTL data; Table S3: Genes showing significantly pleiotropic association with IOP using CAGE eQTL data; Table S4: Genes that showed transcriptome-wide significant associations with IOP.

## References

[1] Armaly M.F., Krueger D.E., Maunder L., Becker B., Hetherington J., Kolker A.E., Levene R.Z., Maumenee A.E., Pollack I.P., Shaffer R.N. (1980). Biostatistical analysis of the collaborative glaucoma study. I. Summary report of the risk factors for glaucomatous visual-field defects. Arch. Ophthalmol..

[2] Chamard C., Villain M., Bron A., Causse A., Bentaleb Y., Pelen F., Baudouin C., Daien V. (2020). Prevalence of Unknown Ocular Hypertension, Glaucoma Suspects, and Glaucoma in Patients Seen in an Ophthalmology Center in France. Ophthalmic Res..

[3] Varma R., Ying-Lai M., Francis B.A., Nguyen B.B., Deneen J., Wilson M.R., Azen S.P., Los Angeles Latino Eye Study Group (2004). Prevalence of open-angle glaucoma and ocular hypertension in Latinos: The Los Angeles Latino Eye Study. Ophthalmology.

[4] Xu L., Wang Y.X., Jonas J.B., Wang Y.S., Wang S. (2009). Ocular hypertension and diabetes mellitus in the Beijing Eye Study. J. Glaucoma.

[5] Gedde S.J., Vinod K., Wright M.M., Muir K.W., Lind J.T., Chen P.P., Li T., Mansberger S.L., American Academy of Ophthalmology Preferred Practice Pattern Glaucoma Panel (2021). Primary Open-Angle Glaucoma Preferred Practice Pattern^®^. Ophthalmology.

[6] Kass M.A., Heuer D.K., Higginbotham E.J., Johnson C.A., Keltner J.L., Miller J.P., Parrish R.K., Wilson M.R., Gordon M.O. (2002). The Ocular Hypertension Treatment Study: A randomized trial determines that topical ocular hypotensive medication delays or prevents the onset of primary open-angle glaucoma. Arch. Ophthalmol..

[7] Heijl A., Leske M.C., Bengtsson B., Hyman L., Bengtsson B., Hussein M., Early Manifest Glaucoma Trial Group (2002). Reduction of intraocular pressure and glaucoma progression: Results from the Early Manifest Glaucoma Trial. Arch. Ophthalmol..

[8] Gordon M.O., Beiser J.A., Brandt J.D., Heuer D.K., Higginbotham E.J., Johnson C.A., Keltner J.L., Miller J.P., Parrish R.K., Wilson M.R. (2002). The Ocular Hypertension Treatment Study: Baseline factors that predict the onset of primary open-angle glaucoma. Arch. Ophthalmol..

[9] Quigley H.A., Broman A.T. (2006). The number of people with glaucoma worldwide in 2010 and 2020. Br. J. Ophthalmol..

[10] Duggal P., Klein A.P., Lee K.E., Iyengar S.K., Klein R., Bailey-Wilson J.E., Klein B.E. (2005). A genetic contribution to intraocular pressure: The beaver dam eye study. Investig. Ophthalmol. Vis. Sci..

[11] Bonovas S., Filioussi K., Tsantes A., Peponis V. (2004). Epidemiological association between cigarette smoking and primary open-angle glaucoma: A meta-analysis. Public Health.

[12] Kang J.H., Pasquale L.R., Rosner B.A., Willett W.C., Egan K.M., Faberowski N., Hankinson S.E. (2003). Prospective study of cigarette smoking and the risk of primary open-angle glaucoma. Arch. Ophthalmol..

[13] Renard J.P., Rouland J.F., Bron A., Sellem E., Nordmann J.P., Baudouin C., Denis P., Villain M., Chaine G., Colin J. (2013). Nutritional, lifestyle and environmental factors in ocular hypertension and primary open-angle glaucoma: An exploratory case-control study. Acta Ophthalmol..

[14] Sanfilippo P.G., Hewitt A.W., Hammond C.J., Mackey D.A. (2010). The heritability of ocular traits. Surv. Ophthalmol..

[15] Asefa N.G., Neustaeter A., Jansonius N.M., Snieder H. (2019). Heritability of glaucoma and glaucoma-related endophenotypes: Systematic review and meta-analysis. Surv. Ophthalmol..

[16] Johnson A.T., Drack A.V., Kwitek A.E., Cannon R.L., Stone E.M., Alward W.L. (1993). Clinical features and linkage analysis of a family with autosomal dominant juvenile glaucoma. Ophthalmology.

[17] Sheffield V.C., Stone E.M., Alward W.L., Drack A.V., Johnson A.T., Streb L.M., Nichols B.E. (1993). Genetic linkage of familial open angle glaucoma to chromosome 1q21-q31. Nat. Genet..

[18] Wirtz M.K., Samples J.R., Kramer P.L., Rust K., Topinka J.R., Yount J., Koler R.D., Acott T.S. (1997). Mapping a gene for adult-onset primary open-angle glaucoma to chromosome 3q. Am. J. Hum. Genet..

[19] Wirtz M.K., Samples J.R., Rust K., Lie J., Nordling L., Schilling K., Acott T.S., Kramer P.L. (1999). GLC1F, a new primary open-angle glaucoma locus, maps to 7q35-q36. Arch. Ophthalmol..

[20] Suriyapperuma S.P., Child A., Desai T., Brice G., Kerr A., Crick R.P., Sarfarazi M. (2007). A new locus (GLC1H) for adult-onset primary open-angle glaucoma maps to the 2p15-p16 region. Arch. Ophthalmol..

[21] Stone E.M., Fingert J.H., Alward W.L., Nguyen T.D., Polansky J.R., Sunden S.L., Nishimura D., Clark A.F., Nystuen A., Nichols B.E. (1997). Identification of a gene that causes primary open angle glaucoma. Science.

[22] Duggal P., Klein A.P., Lee K.E., Klein R., Klein B.E., Bailey-Wilson J.E. (2007). Identification of novel genetic loci for intraocular pressure: A genomewide scan of the Beaver Dam Eye Study. Arch. Ophthalmol..

[23] Lee M.K., Woo S.J., Kim J.I., Cho S.I., Kim H., Sung J., Seo J.S., Kim D.M. (2010). Replication of a glaucoma candidate gene on 5q22.1 for intraocular pressure in mongolian populations: The GENDISCAN Project. Investig. Ophthalmol. Vis. Sci..

[24] Rotimi C.N., Chen G., Adeyemo A.A., Jones L.S., Agyenim-Boateng K., Eghan B.A., Zhou J., Doumatey A., Lashley K., Huang H. (2006). Genomewide scan and fine mapping of quantitative trait loci for intraocular pressure on 5q and 14q in West Africans. Investig. Ophthalmol. Vis. Sci..

[25] Choquet H., Thai K.K., Yin J., Hoffmann T.J., Kvale M.N., Banda Y., Schaefer C., Risch N., Nair K.S., Melles R. (2017). A large multi-ethnic genome-wide association study identifies novel genetic loci for intraocular pressure. Nat. Commun..

[26] Nag A., Venturini C., Small K.S., International Glaucoma Genetics C., Young T.L., Viswanathan A.C., Mackey D.A., Hysi P.G., Hammond C. (2014). A genome-wide association study of intra-ocular pressure suggests a novel association in the gene FAM125B in the TwinsUK cohort. Hum. Mol. Genet..

[27] Van Koolwijk L.M., Ramdas W.D., Ikram M.K., Jansonius N.M., Pasutto F., Hysi P.G., Macgregor S., Janssen S.F., Hewitt A.W., Viswanathan A.C. (2012). Common genetic determinants of intraocular pressure and primary open-angle glaucoma. PLoS Genet..

[28] Bonnemaijer P.W.M., Leeuwen E.M.V., Iglesias A.I., Gharahkhani P., Vitart V., Khawaja A.P., Simcoe M., Hohn R., Cree A.J., Igo R.P. (2019). Multi-trait genome-wide association study identifies new loci associated with optic disc parameters. Commun. Biol..

[29] GTEx Consortium (2017). Genetic effects on gene expression across human tissues. Nature.

[30] Lloyd-Jones L.R., Holloway A., McRae A., Yang J., Small K., Zhao J., Zeng B., Bakshi A., Metspalu A., Dermitzakis M. (2017). The Genetic Architecture of Gene Expression in Peripheral Blood. Am. J. Hum. Genet..

[31] Zhu Z., Zhang F., Hu H., Bakshi A., Robinson M.R., Powell J.E., Montgomery G.W., Goddard M.E., Wray N.R., Visscher P.M. (2016). Integration of summary data from GWAS and eQTL studies predicts complex trait gene targets. Nat. Genet..

[32] Lawlor D.A., Harbord R.M., Sterne J.A., Timpson N., Davey Smith G. (2008). Mendelian randomization: Using genes as instruments for making causal inferences in epidemiology. Stat. Med..

[33] Yang Z., Yang J., Liu D., Yu W. (2021). Mendelian randomization analysis identified genes pleiotropically associated with central corneal thickness. BMC Genom..

[34] Gusev A., Ko A., Shi H., Bhatia G., Chung W., Penninx B.W., Jansen R., de Geus E.J., Boomsma D.I., Wright F.A. (2016). Integrative approaches for large-scale transcriptome-wide association studies. Nat. Genet..

[35] Hartwig F.P., Davies N.M., Hemani G., Davey Smith G. (2016). Two-sample Mendelian randomization: Avoiding the downsides of a powerful, widely applicable but potentially fallible technique. Int. J. Epidemiol..

[36] Pierce B.L., Burgess S. (2013). Efficient design for Mendelian randomization studies: Subsample and 2-sample instrumental variable estimators. Am. J. Epidemiol..

[37] Inoue A., Solon G. (2010). Two-Sample Instrumental Variables Estimators. Rev. Econ. Stat..

[38] Stefansson H., Helgason A., Thorleifsson G., Steinthorsdottir V., Masson G., Barnard J., Baker A., Jonasdottir A., Ingason A., Gudnadottir V.G. (2005). A common inversion under selection in Europeans. Nat. Genet..

[39] Cruts M., Rademakers R., Gijselinck I., van der Zee J., Dermaut B., de Pooter T., de Rijk P., Del-Favero J., van Broeckhoven C. (2005). Genomic architecture of human 17q21 linked to frontotemporal dementia uncovers a highly homologous family of low-copy repeats in the tau region. Hum. Mol. Genet..

[40] Gijselinck I., Bogaerts V., Rademakers R., van der Zee J., van Broeckhoven C., Cruts M. (2006). Visualization of MAPT inversion on stretched chromosomes of tau-negative frontotemporal dementia patients. Hum. Mutat..

[41] De Jong S., Chepelev I., Janson E., Strengman E., van den Berg L.H., Veldink J.H., Ophoff R.A. (2012). Common inversion polymorphism at 17q21.31 affects expression of multiple genes in tissue-specific manner. BMC Genom..

[42] Gharahkhani P., Jorgenson E., Hysi P., Khawaja A.P., Pendergrass S., Han X., Ong J.S., Hewitt A.W., Segre A.V., Rouhana J.M. (2021). Genome-wide meta-analysis identifies 127 open-angle glaucoma loci with consistent effect across ancestries. Nat. Commun..

[43] Gao X.R., Huang H., Kim H. (2019). Genome-wide association analyses identify 139 loci associated with macular thickness in the UK Biobank cohort. Hum. Mol. Genet..

[44] Strunz T., Kiel C., Grassmann F., Ratnapriya R., Kwicklis M., Karlstetter M., Fauser S., Arend N., Swaroop A., Langmann T. (2020). A mega-analysis of expression quantitative trait loci in retinal tissue. PLoS Genet..

[45] Kominato Y., Sano R., Takahashi Y., Hayakawa A., Ogasawara K. (2020). Human ABO gene transcriptional regulation. Transfusion.

[46] Yamamoto F., McNeill P.D., Hakomori S. (1995). Genomic organization of human histo-blood group ABO genes. Glycobiology.

[47] Bennett E.P., Steffensen R., Clausen H., Weghuis D.O., Geurts van Kessel A. (1995). Genomic cloning of the human histo-blood group ABO locus. Biochem. Biophys. Res. Commun..

[48] Li S., Schooling C.M. (2020). A phenome-wide association study of ABO blood groups. BMC Med..

[49] Yamamoto F., Cid E., Yamamoto M., Blancher A. (2012). ABO research in the modern era of genomics. Transfus. Med. Rev..

[50] Hysi P.G., Cheng C.Y., Springelkamp H., Macgregor S., Bailey J.N.C., Wojciechowski R., Vitart V., Nag A., Hewitt A.W., Hohn R. (2014). Genome-wide analysis of multi-ancestry cohorts identifies new loci influencing intraocular pressure and susceptibility to glaucoma. Nat. Genet..

[51] Springelkamp H., Iglesias A.I., Mishra A., Hohn R., Wojciechowski R., Khawaja A.P., Nag A., Wang Y.X., Wang J.J., Cuellar-Partida G. (2017). New insights into the genetics of primary open-angle glaucoma based on meta-analyses of intraocular pressure and optic disc characteristics. Hum. Mol. Genet..

[52] Qian Y., Baisden J.M., Cherezova L., Summy J.M., Guappone-Koay A., Shi X., Mast T., Pustula J., Zot H.G., Mazloum N. (2002). PC phosphorylation increases the ability of AFAP-110 to cross-link actin filaments. Mol. Biol. Cell.

[53] Qian Y., Baisden J.M., Zot H.G., Van Winkle W.B., Flynn D.C. (2000). The carboxy terminus of AFAP-110 modulates direct interactions with actin filaments and regulates its ability to alter actin filament integrity and induce lamellipodia formation. Exp. Cell. Res..

[54] Inoue T., Tanihara H. (2013). Rho-associated kinase inhibitors: A novel glaucoma therapy. Prog. Retin. Eye Res..

[55] Junglas B., Kuespert S., Seleem A.A., Struller T., Ullmann S., Bosl M., Bosserhoff A., Kostler J., Wagner R., Tamm E.R. (2012). Connective tissue growth factor causes glaucoma by modifying the actin cytoskeleton of the trabecular meshwork. Am. J. Pathol..

[56] Bailey J.N., Loomis S.J., Kang J.H., Allingham R.R., Gharahkhani P., Khor C.C., Burdon K.P., Aschard H., Chasman D.I., Igo R.P. (2016). Genome-wide association analysis identifies TXNRD2, ATXN2 and FOXC1 as susceptibility loci for primary open-angle glaucoma. Nat. Genet..

[57] Gharahkhani P., Burdon K.P., Fogarty R., Sharma S., Hewitt A.W., Martin S., Law M.H., Cremin K., Bailey J.N.C., Loomis S.J. (2014). Common variants near ABCA1, AFAP1 and GMDS confer risk of primary open-angle glaucoma. Nat. Genet..

[58] Gao X.R., Huang H., Nannini D.R., Fan F., Kim H. (2018). Genome-wide association analyses identify new loci influencing intraocular pressure. Hum. Mol. Genet..

[59] Liou S.T., Wang C. (2005). Small glutamine-rich tetratricopeptide repeat-containing protein is composed of three structural units with distinct functions. Arch. Biochem. Biophys..

[60] Winnefeld M., Rommelaere J., Cziepluch C. (2004). The human small glutamine-rich TPR-containing protein is required for progress through cell division. Exp. Cell Res..

[61] Wang H., Shen H., Wang Y., Li Z., Yin H., Zong H., Jiang J., Gu J. (2005). Overexpression of small glutamine-rich TPR-containing protein promotes apoptosis in 7721 cells. FEBS Lett..

[62] Vuong T.A., Lee S.J., Leem Y.E., Lee J.R., Bae G.U., Kang J.S. (2019). SGTb regulates a surface localization of a guidance receptor BOC to promote neurite outgrowth. Cell. Signal..

[63] Iglesias A.I., Mishra A., Vitart V., Bykhovskaya Y., Hohn R., Springelkamp H., Cuellar-Partida G., Gharahkhani P., Bailey J.N.C., Willoughby C.E. (2018). Cross-ancestry genome-wide association analysis of corneal thickness strengthens link between complex and Mendelian eye diseases. Nat. Commun..

[64] Khawaja A.P., Cooke Bailey J.N., Wareham N.J., Scott R.A., Simcoe M., Igo R.P., Song Y.E., Wojciechowski R., Cheng C.Y., Khaw P.T. (2018). Genome-wide analyses identify 68 new loci associated with intraocular pressure and improve risk prediction for primary open-angle glaucoma. Nat. Genet..

[65] Khatib Z.A., Inaba T., Valentine M., Look A.T. (1994). Chromosomal localization and cDNA cloning of the human DBP and TEF genes. Genomics.

[66] Hysi P.G., Choquet H., Khawaja A.P., Wojciechowski R., Tedja M.S., Yin J., Simcoe M.J., Patasova K., Mahroo O.A., Thai K.K. (2020). Meta-analysis of 542,934 subjects of European ancestry identifies new genes and mechanisms predisposing to refractive error and myopia. Nat. Genet..

[67] Verbanck M., Chen C.Y., Neale B., Do R. (2018). Detection of widespread horizontal pleiotropy in causal relationships inferred from Mendelian randomization between complex traits and diseases. Nat. Genet..

