# Supplementary figures and images for: Mendelian Randomization and Transcriptome-Wide Association Analysis Identified Genes That Were Pleiotropically Associated with Intraocular Pressure
Source: Genes (Basel). 2023 Apr 30;14(5):1027. doi: 10.3390/genes14051027 (PMC10218376; doi:10.3390/genes14051027)

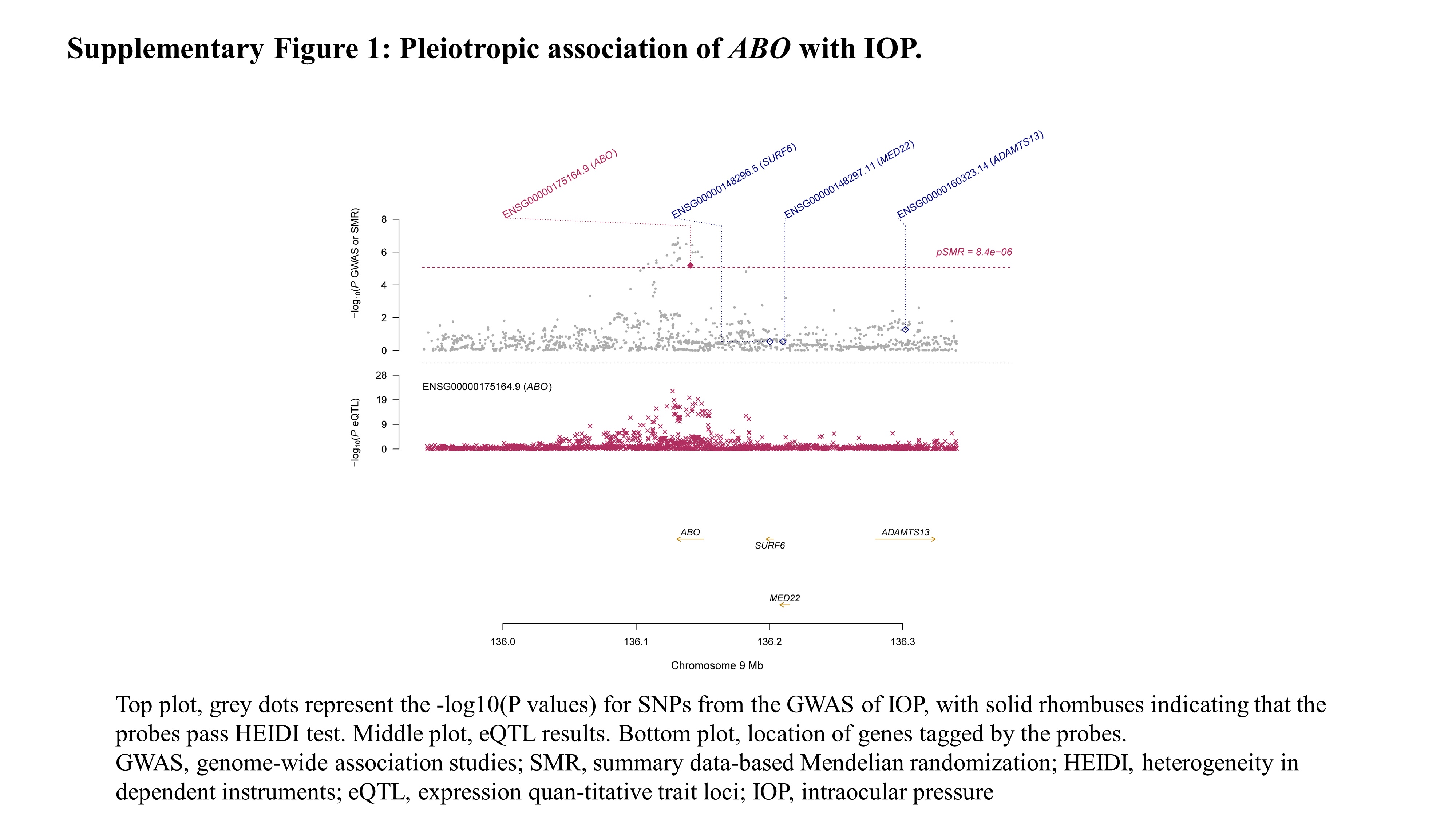

Supplement: Supplementary file 1 [file genes-14-01027-s001.zip › Supplementary_Fgure1.TIF]

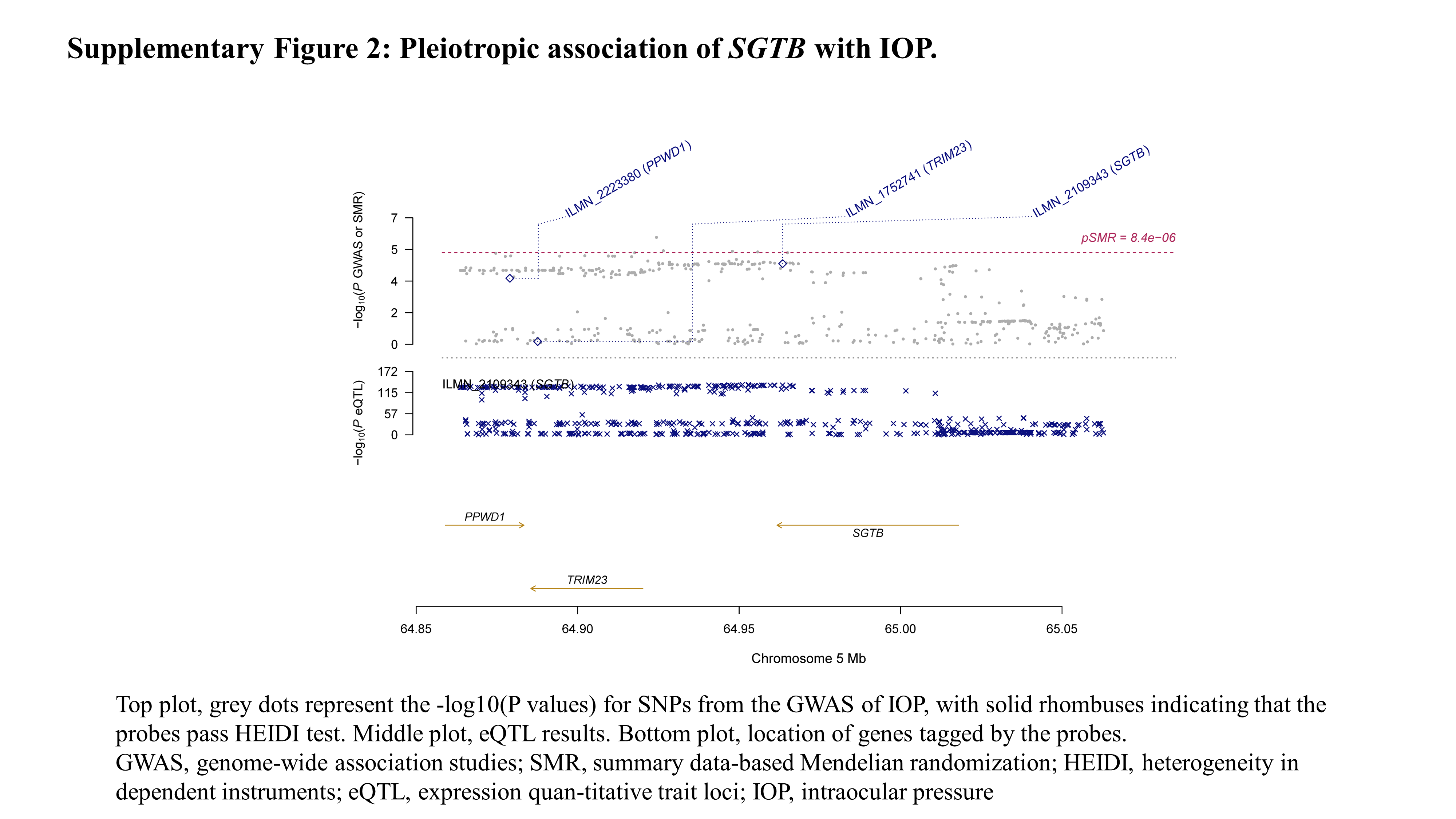

Supplement: Supplementary file 1 [file genes-14-01027-s001.zip › Supplementary_Fgure2.TIF]
